# Laser Ablation for Cancer: Past, Present and Future

**DOI:** 10.3390/jfb8020019

**Published:** 2017-06-14

**Authors:** Emiliano Schena, Paola Saccomandi, Yuman Fong

**Affiliations:** 1 Unit of Measurements and Biomedical Instrumentation, Center for Integrated Research, Università Campus Bio-Medico di Roma, Via Álvaro del Portillo, 21, Rome 00128, Italy; 2 IHU (Institute of Image-Guided Surgery), S/c Ircad, Strasbourg 67091, France; paola.saccomandi@ihu-strasbourg.eu; 3 Department of Surgery, City of Hope, Duarte-Main Campus, 1500 East Duarte Road, Duarte, CA 91010, USA; yfong@coh.org

**Keywords:** laser ablation, cancer therapy, local cancer therapy, thermal ablation, thermography

## Abstract

Laser ablation (LA) is gaining acceptance for the treatment of tumors as an alternative to surgical resection. This paper reviews the use of lasers for ablative and surgical applications. Also reviewed are solutions aimed at improving LA outcomes: hyperthermal treatment planning tools and thermometric techniques during LA, used to guide the surgeon in the choice and adjustment of the optimal laser settings, and the potential use of nanoparticles to allow biologic selectivity of ablative treatments. Promising technical solutions and a better knowledge of laser-tissue interaction should allow LA to be used in a safe and effective manner as a cancer treatment.

## 1. Introduction

Many ablative techniques are being proposed as alternatives to traditional resectional surgery. These include laser ablation (LA), radio-frequency ablation, microwave ablation, high intensity focused ultrasound treatments, and cryosurgery. All these techniques hold the promise of cancer killing while sparing normal tissue. Ablative therapy can also be delivered in a minimally invasive manner, allowing less pain and shorter recovery time. Among the mentioned techniques, LA shows the attractive possibility of being guided through a flexible and small fiber to targets in deep-lying organs.

The first application of laser in surgery dates shortly after its invention, when in 1961 Salon and coauthors investigated its potential as a clinical tool [1]. In the 1980s, the first preclinical and clinical testing of lasers as ablative tools for brain cancer, gastrointestinal tumors (liver and pancreas), and prostate cancer occurred [2,3]. Many different lasers have been proposed for use in surgery. This article reviews the state of the art of the lasers most used in ablative procedures for cancer removal: with particular attention on the characteristics of various lasers, on the factors which influence the treatment outcome, and on the emerging solutions proposed to improve the outcomes of LA.

## 2. Basic Components of a Laser and Factors of Influence on the Laser Effect on Tissue

LA is performed by using a laser and a medium which transports the laser light inside the tissue. The laser, which consists of a power source, a lasing medium, and reflecting mirrors, provides a monochromatic light (the light is emitted at a specific wavelength), whose wavelength defines the properties of the laser and the interaction with biological tissue. The medium is usually a small diameter (0.2–0.8 mm) flexible optical fiber that transports the laser light inside deep organs. Laser-tissue interaction can be described by three phenomena: scattering, reflection, and absorption. The light absorbed by tissue is converted into heat. Prolonged exposure of tumor cells at temperatures ranging from 45 °C to 55 °C or short exposure at temperature higher than 60 °C causes irreversible cell damage [4]. Complex mathematical descriptions, based on Arrhenius rate analysis, allow for estimating the cell death as a function of both temperature and exposure time [5].

**Figure 1:**
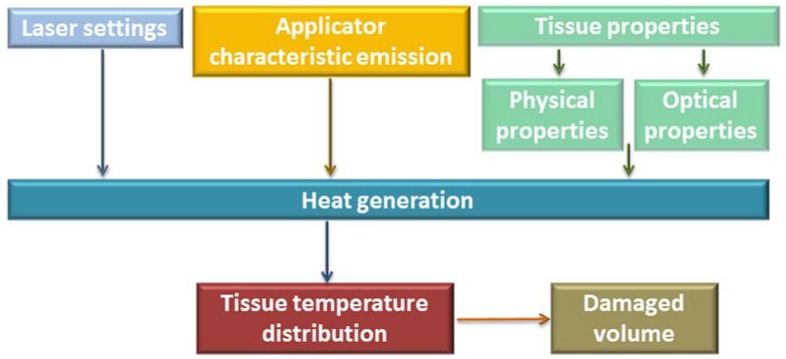
Schematic representation of the factors of laser ablation influencing the volume of tissue destruction.

Heat generation in the tissue, hence the effect of LA, is influenced by a number of factors: laser light wavelength, laser settings (laser power, laser energy, and treatment time), physical properties of the tissue, and the emission characteristics of the optical applicator [6], see Figure 1.

In order to destroy the tumor without damaging healthy surrounding tissue or minimizing this unwanted effect, all the mentioned factors must be taken into account during the treatment. In particular, a very important parameter used to describe how the laser light is absorbed by the tissue is the absorption length, defined as the tissue depth needed to absorb 63% of the incident light. The absorption length is specific for different laser types (laser light wavelength). It is also related to the optical characteristics of the specific tumor and the surrounding healthy tissue [7]. Lasers with wavelengths strongly absorbed by tissue are employed for superficial treatment. Conversely, in order to treat deep tumors, a high optical penetration depth is required.

The choice of laser settings is related to the specific effects desired during the procedure. LA can be performed in continuous mode or in pulsed mode. In continuous mode, low laser power (ranging from 2 W or 3 W up to 30 W), and long treatment time (from a few minutes to more than 20 min) are usually employed [8]. In pulsed mode, in which the laser energy is released intermittently in a series of pulses rather than continuously, higher laser power (>100 W) is used. It must be noted that the tissue temperature increase (hence the amount of damaged volume) is not a linear function with respect to the laser settings [9]. Thus, tissue temperature and damage volume plateau with increasing treatment time and laser power.

The emission characteristics of the optical applicator play a great role on the geometry of the damaged tissue. Applicators called “bare-fiber” were employed during the first applications of LA. Basically, they are an optical waveguide with an emitting distal end. Appropriate designs and manufacturing of the emitting surface of the applicators allow for reducing the power density and the temperature on their surface, and allow for controlling the tissue geometry damaged [10]. Sapphire-tipped fibers were introduced to avoid carbonization around the fiber tip [11] in order to penetrate more deeply inside the tissue because charred tissue limits light penetration and tissue necrosis [12]. Then other applicators were designed and validated, such as the cylindrical fiber tip [13] and zebra applicators [14]. Moreover, several solutions based on the development of cooled tip applicators or on the use of multiple bare fibers have been proposed to obtain large and controlled damaged volumes [15,16,17,18].

Laser use brings about specific safety concerns. LA is performed by lasers emitting light at a power higher than 0.5 W. Medical lasers are therefore Class IV lasers according to the ANSI (American National Standards Institute) standard (ANSI Z136.1 and Z136.3 combination set: “Safe Use of Lasers and Safe Use of Lasers in Health Care Facilities”). The high power of light emitted by Class IV lasers can damage the eye and skin. As a consequence, administrative, engineering, and procedural measures are used to control laser hazards. All the personnel involved in the LA have to be qualified. All should wear protective eyewear for the specific wavelength and optical density used. Moreover, the access to the room during LA should be controlled, and laser hazard signs indicating the class, power, and mode of operation of the laser should be posted.

## 3. Lasers in Surgery

**Figure 2:**
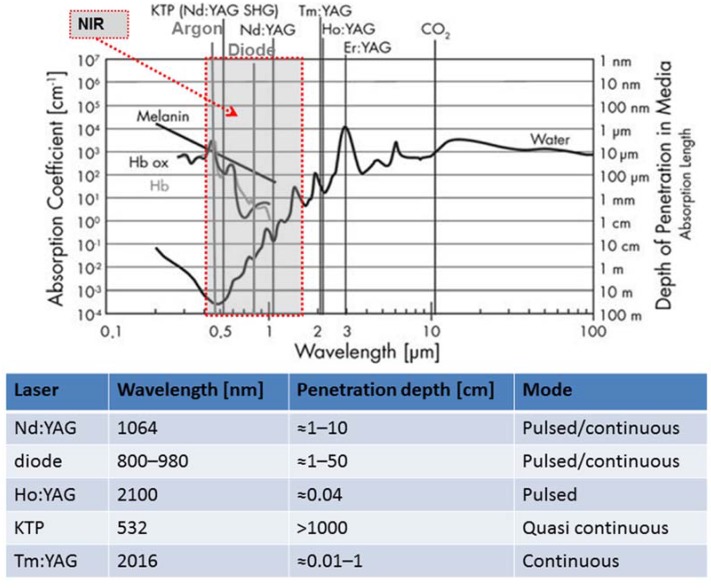
Wavelength, penetration depth, and modality of work of widely employed medical lasers. The absorption spectra of melanin, hemoglobin, and water are also shown.

As described in the previous section, the effects of laser light on tissue depend on the laser operation mode and on its light wavelength. As a consequence, many different lasers have been used to ablate tumors, in order to obtain different effects. They differ mainly according to their wavelength, hence absorption length (Figure 2).

When deep penetration is required, lasers emitting infrared light are employed. Diode lasers, with wavelengths of 800–980 nm and Nd:YAG (neodymium-doped yttrium aluminium garnet; Nd:Y3Al5O12) lasers with a wavelength of 1064 nm have an absorption length of approximately 10 cm, as shown in Figure 2. The KTP:YAG laser (KTP stands for potassium-titanyl-phosphate) emits at 532 nm, and is highly absorbed by hemoglobin but deeply penetrates in water. This difference is pointed in the plot shown in Figure 2. The absorption of light is limited to three important components of biological tissue because the analysis of specific organs will result in difficulties, the data are incomplete, and the experimental data regarding absorption values show high dispersion [7]. Superficial treatments were performed with a CO_2_ laser (10,600 nm), Thulium (2016 nm), and Ho:YAG (2100 nm) with lower penetration depths (from around 10 μm to almost 1 mm).

Nd:YAG, Ho:YAG, and diode lasers were the original lasers deployed in clinical practice. The Nd:YAG laser (1064 nm) is usually used in continuous mode. It has been for decades the most widely used laser because the high penetration of its wavelength is optimal in the treatment of several tumors. The ablation is mostly performed with bare or cylindrical applicators which allows for ablation zones of up to 15 mm and 50 mm diameter, respectively [19]. The use of cooled applicator allows for improving the radial temperature distribution, avoiding carbonization, and using higher laser power.

Table 1 reports a number of applications of Nd:YAG lasers in surgery.

Hepatocellular carcinoma (HCC) and liver metastases were the most commonly treated cancers by Nd:YAG lasers. These treatments are performed with low power, measured in Watts, and the time of treatment, measured in minutes (e.g., 5 W and 6–12 min [20]). Laser power can be increased to 30 W–40 W with cooled applicators [41]. Different groups used this laser for liver metastases [21] with good results in terms of survival rate and complications [23,24,25,26]. Large liver metastases have been treated with modified techniques consisting of “pull back” of the applicator or the use of multiple applicators [22].

Premalignant lesions and early stages of penile cancer carcinoma have been treated since the 1980s. The indication for the use of LA in this clinical setting is superficial penile cancer (either Tis or T1 disease). Contraindications to laser therapy include tumors with >6 mm depth invasion and T2 tumors [42]. Recently several studies focused on the efficacy of Nd:YAG lasers on penile cancer [29,30] and on the combination of Nd:YAG and CO_2_ lasers [27,28], with good results in terms of local recurrence and satisfaction after the treatment, as well as good functional and cosmetic outcomes [27,43].

During the 1980s and 1990s, bladder cancer was treated by Nd:YAG lasers with high power and short time pulses [31,33,34,44]. The main risk is the perforation of the bowel or bladder [45], in particular at high laser power (>50 W) [46], although this has also been reported at 35 W [32]. This laser has been also used for the ablation of cervical lymph node metastases from papillary thyroid carcinoma with good results in terms of technical success (100% of the lymph nodes) and on complications (there were no major complications) [40]. Despite promising results, the use of Nd:YAG lasers on the treatment of bladder cancer has been abandoned, with the introduction of alternative lasers (see below).

Nd:YAG LA has been used as palliative treatments for several other cancers, e.g., colorectal [47,48], pancreas neuroendocrine tumors [39], lung [35,49], glioblastoma [36], osteoid osteoma [37], renal [38], ureteral tumors [50], and breast cancer [51]. These uses have generally been delivered at power settings of 5 W and a few minutes of application, or at high (50 W) power with short pulses of 1–3 s.

The Ho:YAG laser operates in pulsed mode at a wavelength of 2100 nm. Since the 1990s, it has replaced the Nd:YAG laser for the treatment of superficial bladder cancer [52,53]. Treatments are performed at different frequencies (5 Hz–40 Hz), energy per pulse (0.5 J–2.2 J), and power (4 W–40 W) [52,53,54,55,56,57,58,59,60,61], and show peri- and post-operative complication rates lower when compared to conventional transurethral resection (Table 2). In urology, this laser has also been employed to treat upper urinary tract tumors with settings similar to the ones employed during bladder ablation [62].

Laser diodes are replacing the Nd:YAG laser because they are more compact and portable (weighting less than 10 kg), less expensive, and deliver wavelengths between 800 nm and 980 nm with tissue penetration similar to that obtained by Nd:YAG lasers. Diode lasers have been largely employed on prostatic tumors with very good results in terms of complications and tumor recurrence (Table 3). The treatment is performed at different wavelengths (805 nm, 830 nm, or 980 nm) and the amount of damaged tissue is controlled with ultrasound, with temperature monitoring by fluoroptic thermal probes [63,64,65].

Laser diodes (980 nm) have also been used for metastatic brain tumors using a temperature feedback obtained by MR (Magnetic Resonance)-based thermometry, with reasonable preliminary results on four patients in terms of both tumor recurrence and complications [67]. Osteoid osteoma has been treated by a diode laser (805 nm) with good results in terms of recurrence (only six recurrences in a cohort of 114 patients and all were treated successfully with a second application) [66]. Because of the increasing detection of small breast cancer due to the widespread use of mammography, the diode laser (805 nm) is also being investigated for the treatment of small tumors with the use of temperature feedback [68,69]. It is also being explored for the treatment of hepatic metastases from colorectal cancer using a wavelength of 810 nm [70].

## 4. New Solutions to Guide Laser Ablation

The most promising emerging solutions in terms of the potential clinical impact on LA aim at controlling with high accuracy the amount of damaged tissue or at obtaining a more selective tumor treatment that does not damage the surrounding healthy tissue. Recent efforts are devoted to the development of Hyperthermal Treatment Planning (HTP) tools, to the improvement of new solutions for real time thermometry, and to the use of tumor targeted nanoparticles. In this section, the basis and the most significant challenges of these three promising solutions will be described.

HTP tools aim at establishing the treatment settings that maximize the thermal treatment quality. HTPs model the interaction between the energy delivered by the thermal treatment and the tissue, in order to obtain a prediction of the tissue temperature distribution and therefore the amount of damaged tissue volume. As described in [71], the simulations can be divided in three main steps: (1) the first step is the generation of the patient model. This is aimed at obtaining a description of the geometry and of the physical properties of the tissue undergoing the treatment. This first step is crucial because the geometry and characteristics of the tissue strongly influence the interaction between the tissue and the energy delivered to treat the tumor (i.e., laser light in the case of LA treatment); (2) the second step is focused on the calculation of the amount of power absorbed by the tissue. Obviously the models employed depend on the kind of device used to induce the hyperthermia. In LA, the simulation is aimed at calculating the light distribution within the tissue. This task is usually performed using the Monte Carlo simulation and requires the knowledge of the tissue optical properties at the used laser wavelength and the emission modality of the applicator; (3) the third step provides the tissue temperature distribution. The model most widely used to perform this prediction is the Pennes’ equation. The accurate prediction of temperature can improve the treatment outcomes.

The importance of HTP (hyperthermal treatment planning) tools in current clinical settings is confirmed by the recent decision of the European Society for Hyperthermic Oncology to include HTP in their quality assurance guidelines for deep hyperthermia [72], and by the recent development of several commercial treatment planning packages (e.g., the Sigma -Hyperplan system, VEDO, Semcad X, and Alba HTPS) and flexible software packages [73,74]. HTP tools have been clinically evaluated and validated [75,76]. Recently, Hyperplan predicted both the occurrence of discomfort and its location in a cohort of 30 patients with an error of the temperature prediction lower than 4 °C [77]. HTP tools have been also used for improving the safety and effectiveness of local hyperthermal treatments combined with radiotherapy and chemotherapy [78,79]. In spite of the HTPs limitations in the accurate prediction of the temperature distribution, they have demonstrated marked improvements over the last few years, so their integration into the clinical workflow is gaining acceptance [80]. In addition, temperature feedback obtained by thermometric techniques could correct HTP prediction during the treatment.

The importance of temperature monitoring during LA can be motivated by considering that the amount of damaged tissue depends on both the tissue temperature map and the exposure time [81]; therefore the knowledge in real time of the tissue temperature may be particularly beneficial for the optimization of laser settings applied during treatment. Thermometric techniques can be divided in two categories: invasive techniques and non-invasive techniques [82].

Among the invasive thermometric techniques, the most largely employed transducers are thermistors, thermocouples, and fiber optic-based sensors. Their use has been investigated in many recent in vivo and ex vivo cancer thermal treatment studies [83,84,85,86] and on different organs [87,88]. They allow for real time temperature monitoring with good spatial resolution, and quite good (thermocouples) or good (thermistors) accuracy. Their main drawbacks are related to their intrinsic invasiveness, and measurement only at a single point. There can also be measurement errors due to the strong light absorption of the wires of the thermocouple [89,90,91] and due to the high heat conduction for both thermocouples and thermistors.

Two kinds of transducers based on fiber optic technology are employed in this field: Fiber Bragg Grating (FBG) sensors and fluoroptic sensors. These sensors have been introduced in this field more recently than thermocouples and thermistor [63,92,93]. Their main advantages are related to due to their immunity from electromagnetic fields and their MR-compatibility, which allows for using this sensor during MR-guided procedures [94]. Their small size and flexibility, short response time, good spatial resolution, and good accuracy (≈0.2 °C) are also assets. Their main drawbacks are related to their invasiveness, and measurement only at a single point for flouroptic sensors. Moreover, FBGs are sensitive to the strain that can produce measurement errors during in vivo trials caused by the respiratory movements of the patients [95]. Temperature probes embedding FBGs within a needle have been proposed to tackle this problem [96,97]. Regarding the fluoroptic sensor, the error caused by laser light absorption cannot be considered as negligible [91].

The most promising non-invasive thermometric methods are MR-based thermometry and CT (Computed Tomography)-based thermometry.

Basically, MR-thermometry is founded on the dependence of a number of MR parameters on temperature [98]. After a series of experiments on phantoms, ex vivo tissues, and on in vivo animal models [99], MR thermometry has been employed during LA of HCC and liver tumors , prostate cancer, and metastases during the last decade [100,101,102]. Recent studies have shown the possibility of obtaining good spatial and temporal resolution and good precision [103].

CT-thermometry was first investigated during the 1970s [104], but investigations were discouraged by the limitation of the CT scan in terms of reproducibility and stability. In the last decade, the improvements of CT scanners have encouraged a number of groups to use this method in thermal treatment. During the last few years this technique has been mainly employed during ex vivo experiments and on phantoms [105,106,107,108,109,110]. Although laser ablation guided by non-invasive thermometry is in its infancy, recent technical solutions are helping to increase the number of studies in animal models and in humans.

The main advantages of these two non-invasive techniques are related to the non-invasiveness and to the possibility of obtaining a tridimensional temperature distribution. The main disadvantages of MR-thermometry are related to the cost of the MR scan, cost of custom made sequences to obtain good thermal sensitivity, and the hazards of working in an MR environment; the main drawback of the CT-based thermometry is related to the use of ionizing radiation.

Finally, an emerging solution which is noteworthy is the use of nanoparticles in the photothermal ablation of cancer. The aim of this solution is to improve the selectivity of the treatment in order to destroy the tumor while preserving the integrity of the healthy surrounding tissue. The basis of this therapy is that materials that highly absorb light can be designed and delivered specifically to the tumor cells. The subsequent application of light will then cause specific thermal killing to nanoparticle tagged tumor cells.

Gold based nanoparticles have been designed and absorb light in the near-infrared (NIR) region where water and hemoglobin show high transmissivity (as shown in Figure 2). If the nanoparticles are selectively accumulated in the tumor, the light will be mostly absorbed by the tumor only. As a consequence, the absorbed light that is converted into heat energy causes a temperature increase localized in the target. This specificity depends on the geometry, morphology, and surface charge of the nanoparticles; therefore several kinds of gold nanoparticles have been designed for photothermal ablation to optimize the absorption and selectivity (e.g., nanorods, nanoshells, branched nanoparticles, and nanocages) [111]. These nanoparticles have been used in several cancer models (e.g., breast cancer, pancreatic cancer) [112,113,114]. The comparison between the effects on cells in the absence of nanoparticles and on cells with nanoparticles has been performed to assess the efficacy of this technique [112,115]. For instance, El-Sayed et al. noted that in the absence of nanoparticles, the cells did not experience destruction up to a laser power density of 76 W/cm^2^; on the other hand, benign cells with nanoparticles were destroyed at 57 W/cm^2^, and for malignant cells it occurred at a lower value (25 W/cm^2^) [116]. Recently, this technique has been evaluated in vivo in animal models. The efficacy of in vivo treatment has correlated to the findings that nanoshell-treated tumors were noted to experience a temperature increase higher than that for the nanoshell-free controls (37.4 ± 6.6 °C vs. 9.1 ± 4.7 °C) [117].

Clearly, the use of nanoparticles in this treatment approach is in its infancy. The early promising results bring expectations that this approach may have an important future clinical impact. Further improvements and successful introduction in therapy require a proper evaluation and understanding of their interactions with biological entities and their potential for inadvertent toxicities [118,119].

## 5. Discussion

LA is becoming a valid alternative to surgical resection. The ultimate goal of LA is to reduce the suffering related to specific cancers and to improve outcomes. After the tumor localization and the identification of its features (geometry, contours, histology), there are two main challenges in LA: an accurate placement of the applicator in the tumor, and accurate treatment planning and monitoring. New HTP tools and monitoring tools are beginning to overcome some of these challenges, and they are gaining widespread attention and broad clinical acceptance as techniques for improving the safety and outcomes of thermal treatments.

The current landscape of LA is changing rapidly, with new and exciting developments. Among others, emerging solutions and developments which are noteworthy are: the recent evolution in the use of new lasers with different wavelengths and modes of operation, and equipment (e.g., custom applicators) are leading to promising results in terms of treatment selectivity; the improved understanding of the laser-tissue interactions is used to increase the accuracy of computational models for HTP tools for planning patient-specific treatments; the improvement in precision and accuracy of tridimensional non-invasive thermometry and the increasing interest in multi-point temperature probes based on FBG technology are gaining widespread attention for the real time monitoring of the effects of LA; and lastly, the progress in targeting nanoparticles to tumor cells as well as the possibility to specifically tune the laser to the surface plasmon resonance frequency of the nanoparticles are paving the way for the advent of targeted heating. For the promise of this technology to be realized, new solutions, such as HTP tools, thermometry, and the advancement of nanotechnology in medicine, have to be further improved and translated for clinical use. This requires a continued and close research collaboration between interdisciplinary groups involving clinical experts, physicists, bioengineering, and material scientists.

## Figures and Tables

**Table 1:** Nd:YAG laser for tumor ablation (y = years; m = months; SR = Survival Rate; HCC = Hepatocellular carcinoma; met = metastases; P = laser power; t = treatment time).

Author	Tumor (Number of Patients)	Diameter of Tumor	Applicator	Laser Settings	Follow Up/Complications
Pacella et al., 2001 [20]	HCC (74)	0.8–4 cm	Bare fiber	P = 5 W t = 6–12 min	SR: 99%, 68%, and 15% at 1, 3, and 5 y
Vogl et al., 2002 [21]	Malignant liver tumor (899)	---	Bare or Cooled fiber	P = 4–5 W bare P = 35–45 W cooled fiber t = 3–28 min	0.1% death 13% of overall complications
Vogl et al., 2013 [22]	Malignant liver tumor (401)	<5 cm	Cooled fiber	P mean for each applicator 34 W	SR: 86.5% and 33.4% at 1 and 5 y
Dick et al., 2003 [23]	Primary and secondary liver tumor (35)	---	Cooled fiber	P = 25 W t = 10–30 min	Mean SR: HCC: 14.6 m (for HCC), 15.2 m (for met) Carcinoid (all patients alive from 1 to 47 m post ablation)
Pech et al., 2007 [24]	Colorectal liver met (66)	≤5 cm	Cooled diffuser tip fiber	P = 10 W per cm diffusor length t = 15–37 min	Median of SR 23 m Major complications rate 2.3%
Ritz et al., 2007 [25]	Colorectal liver met (56)	≤5 cm	Cooled diffuser fiber tip	P = 24–30 W t = 20–28 min	After 6 m follow-up, tumor recurrence in 6 patients Morbidity rate 21.4%
Christophi et al., 2004 [26]	Colorectal liver met (80)	<10 cm	Bare fiber	P = 2–4 W	Overall complications 16%
Windahl et al., 2004 [27]	Penile cancer (67)	<3 cm	---	---	Median follow up: 42 m Local recurrence rate 19%
Lont et al., 2005 [28]	Penile cancer (257)	<3 cm	---	---	Median follow up 106 m Local recurrence rate 37.5%
Meijer et al., 2007 [29]	Penile cancer (44)	---	---	P = 25–35 W	Follow up 3 m–16 y Local disease: 48% of the patients
Schlenker et al., 2010 [30]	Penile cancer (54)	---	Cooled bare fiber	P = 30–50 W t = 60–150 s	Local recurrence: 42%, mean time to local recurrence 53 m
Beer et al., 1989 [31]	Bladder cancer (252)	---	Bare fiber	P = 40–50 W t per pulse = 3–4 s	Total complications 15% Only 1 bladder perforation
J. Ruiz-Tovar et al., 2008 [32]	Bladder cancer (1)	---	Bare fiber	P = 35 W t per pulse = 2 s	Bladder perforation
Beisland et al., 1985 [33]	Bladder cancer (100)	---	Bare fiber	P = 45–50 W t per pulse < 4 s	1 bowel perforation, 2 severe bleeding
Kardos et al., 1994 [34]	Bladder cancer (116)	7 mm of average	---	P = 30–40 W t (per pulse) = 2–3 s	No major complications
Cavaliere et al., 1994 [35]	Breast cancer (1585)			P = 20–30 W t (per pulse) = 4–5 s	Major limitation: rapid regrowth of the tumor
Schwarzmaier et al. 2006 [36]	Glioblastoma (16)	>20 mm	Diffuser tip	P = 6 W	Overall survival longer than those reported from natural history or after chemotherapy
Streitparth et al., 2009 [37]	Osteoid osteoma (1)	5 mm	Bare fiber	P = 2.3 W t = 11 min	---
Dick et al., 2002 [38]	Renal tumor (9)	---	Cooled Bare fiber	P = 25 W t = 10–30 min	Two minor and one major complications
Di Matteo et al. 2013 [39]	Neuroendocrine Pancreatic tumor (1)	---	Bare fiber	P = 4 W t = 5 min	---
Mauri et al., 2016 [40]	cervical lymph node met (24)	---	---	1 or 2 fibers, P = 3–4 W t = 5–10 min	No major complications; 2 minor complications (8.3%).

**Table 2:** Ho:YAG laser for tumor ablation (E= energy delivered by the treatment; P = laser power; f = frequency of the pulse).

Author	Tumor (Number of Patients)	Diameter of Tumor	Applicator	Laser Settings	Follow Up/Complications
Syed et al., 2001 [55]	Bladder (41)	<1 cm	Bare fiber	E = 0.5–1.0 J f = 5–10 Hz	No complications
Razvi et al., 1995 [53]	Bladder (25)	<1 cm	Bare fiber	E = 0.5–1.0 J P = 4–7.2 W f = 8–14 Hz	No complications
Das et al., 1998 [56]	Bladder (23)	---	Bare fiber	---	1× recatheterization
Johnson, 1994 [52]	Bladder (15)	2–15 mm	Bare fiber	E = 1 J P = 10 W f = 10 Hz	No complications
Jonler et al., 2004 [57]	Bladder (52)	2–30 mm	Bare fiber	E = 1 J P = 40 W f = 40 Hz	Recurrence
Hossain et al., 005 [58]	Bladder (30)	<40 mm	Bare fiber	E = 0.5–1.2 J f = 10–12 Hz	Recurrence
Zhu et al., 2008 [59]	Bladder (101)	---	Bare fiber	E = 1.5–2.2 J P = 20–40 W f = 15–20 Hz	---
Xishuang et al., 008 [61]	Bladder (64)	---	Bare fiber	E = 1.5 J P = 30 W f = 20 Hz	---
Wong et al., 2013 [60]	Bladder (54)	<30 mm	Bare fiber	E = 0.6–0.8 J f = 10–15 Hz	Recurrence
Matsuoka et al., 2003 [62]	Upper urinary tract (30)	5–30 mm	Bare fiber	E = 0.5–1 J P = 15–40 W f = 5–20 Hz	Recurrence

**Table 3:** Diode laser for tumor ablation (met = metastases; P = laser power; t = treatment time).

Author	Tumor (Number of Patients)	Diameter of Tumor	Applicator	Laser Settings	Follow Up/Complications
Atri et al., 2009 [65]	Prostatic carcinoma (1)	16 mm	1 bare fiber	Two lasers at P = 15–2 W, t = 12 min	Necrotic tissue in targeted area
Amin et al., 1993 [64]	Prostatic carcinoma (1)	---	3 applicators	P = 2 W t = 500 s	Biopsies confirmed the presence of necrosis
Linder et al., 2009[63]	Prostatic carcinoma (12)	---	1 or 2 applicators	---	67% of patients free of tumor in the target at 6 m
Gangi et al., 2007 [66]	Osteoid osteoma (114)	<24 mm	Bare fiber	P = 2 W t ≤ 600 s	6 recurrence, 1 unsuccessful treatment
Carpentier et al., 2008 [67]	Metastatic intracranial tumor (4)	<3 cm	Light-diffusing tip	P = 15 W	No tumor recurrence
Dowlatshahi et al., 2002 [68]	Breast tumor (54)	5–23 mm	---	P = 5 W	Complete destruction of 93% of the tumors
Haraldsdóttir et al., 2008 [69]	Breast tumor (54)	---	Bare fiber	P = 3 W	Small skin necrosis in two patients
Gillams et al., 2000 [70]	Hepatic met (69)	---	Bare fiber	P = 2–2.7 W for each fiber t = 440 s	LA improves survival in patients with inoperable but limited liver met.

## References

[1] Solon L.R., Aronson R., Gould G. (1961). Physiological implications of laser beams. Science.

[2] Bown S.G. (1983). Phototherapy in tumors. World J. Surg..

[3] Muschter R., Hofstetter A. (1995). Interstitial laser therapy outcomes in benign prostatic hyperplasia. J. endourol..

[4] Izzo F. (2003). Other Thermal Ablation Techniques: Microwave and Interstitial Laser Ablation of Liver Tumors. Ann. Surg. Oncol..

[5] Stafford R.J., Fuentes D., Elliott A.A., Weinberg J.S., Ahrar K. (2010). Laser-induced thermal therapy for tumor ablation. Crit. Rev. Biomed. Eng..

[6] Muller G.J., Roggan A. (1995). Laser-induced interstitial thermotherapy.

[7] Jacques S.L. (2013). Optical properties of biological tissues: A review. Phys. Med. Biol..

[8] Nikfarjam M., Christophi C. (2003). Interstitial laser thermotherapy for liver tumours. Br. J. Surg..

[9] Saccomandi P., Schena E., Caponero M.A., Di Matteo F.M., Martino M., Pandolfi M., Silvestri S. (2012). Theoretical Analysis and Experimental Evaluation of Laser-Induced Interstitial Thermotherapy in ex Vivo Porcine Pancreas. IEEE Trans. Biomed. Eng..

[10] Schwarzmaier H.-J., Goldbach T., Ulrich F., Schober R., Kahn T., Kaufmann R., Wolbarsht M.L., Cerullo L.J., Heiferman K.S., Liu H., Podbielska H., Wist A.O., Zamorano L.J. (1994). Improved laser applicators for interstitial thermotherapy of brain structures. Proceedings of the Clinical Applications of Modern Imaging Technology II.

[11] Möller P.H., Lindberg L., Henriksson P.H., Persson B.R.R., Tranberg K.-G. (1995). Interstitial laser thermotherapy: Comparison between bare fibre and sapphire probe. Lasers Med. Sci..

[12] Sturesson C. (1998). Interstitial laser-induced thermotherapy: Influence of carbonization on lesion size. Lasers Surg. Med..

[13] Heisterkamp J., van Hillegersberg R., Sinofsky E., Ijzermans J.N.M. (1997). Heat-resistant cylindrical diffuser for interstitial laser coagulation: Comparison with the bare-tip fiber in a porcine liver model. Lasers Surg. Med..

[14] Mensel B., Weigel C., Hosten N. (2006). Laser-Induced Thermotherapy. Minimally Invasive Tumor Therapies.

[15] Germer C.T., Albrecht D., Roggan A., Buhr H.J. (1998). Technology for In Situ Ablation by Laparoscopic and Image-Guided Interstitial Laser Hyperthermia. Surg. Innov..

[16] Germer C.T., Albrecht D., Isbert C., Ritz J., Roggan A., Buhr H.J. (1999). Diffusing Fibre Tip for the Minimally Invasive Treatment of Liver Tumours by Interstitial Laser Coagulation (ILC): An Experimental Ex Vivo Study. Lasers Med. Sci..

[17] Steger A.C., Lees W.R., Shorvon P., Walmsley K., Bown S.G. (1992). Multiple-fibre low-power interstitial laser hyperthermia: Studies in the normal liver. Br. J. Surg..

[18] Saccomandi P., Schena E., Giurazza F., Del Vescovo R., Caponero M.A., Mortato L., Panzera F., Cazzato R.L., Grasso F.R., Di Matteo F.M. (2014). Temperature monitoring and lesion volume estimation during double-applicator laser-induced thermotherapy in ex vivo swine pancreas: A preliminary study. Lasers Med. Sci..

[19] Muralidharan V., Malcontenti-Wilson C., Christophi C. (2002). Interstitial laser hyperthermia for colorectal liver metastases: The effect of thermal sensitization and the use of a cylindrical diffuser tip on tumor necrosis. J. Clin. Laser Med. Surg..

[20] Pacella C.M., Bizzarri G., Magnolfi F., Cecconi P., Caspani B., Anelli V., Bianchini A., Valle D., Pacella S., Manenti G. (2001). Laser Thermal Ablation in the Treatment of Small Hepatocellular Carcinoma: Results in 74 Patients. Radiology.

[21] Vogl T.J., Farshid P., Naguib N.N.N., Darvishi A., Bazrafshan B., Mbalisike E., Burkhard T., Zangos S. (2014). Thermal ablation of liver metastases from colorectal cancer: Radiofrequency, microwave and laser ablation therapies. Radiol. Med..

[22] Vogl T.J., Freier V., Nour-Eldin N.-E.A., Eichler K., Zangos S., Naguib N.N.N. (2013). Magnetic Resonance–Guided Laser-Induced Interstitial Thermotherapy of Breast Cancer Liver Metastases and Other Noncolorectal Cancer Liver Metastases. Investig. Radiol..

[23] Dick E.A., Joarder R., De Jode M., Taylor-Robinson S.D., Thomas H.C., Foster G.R., Gedroyc W.M. (2003). MR-guided Laser Thermal Ablation of Primary and Secondary Liver Tumours. Clin. Radiol..

[24] Pech M., Wieners G., Freund T., Dudeck O., Fischbach F., Ricke J., Seemann M.D. (2007). MR-guided interstitial laser thermotherapy of colorectal liver metastases: Efficiency, safety and patient survival. Eur. J. Med. Res..

[25] Ritz J.-P., Lehmann K.S., Zurbuchen U., Wacker F., Brehm F., Isbert C., Germer C.T., Buhr H.J., Holmer C. (2007). Improving laser-induced thermotherapy of liver metastases—Effects of arterial microembolization and complete blood flow occlusion. Eur. J. Surg. Oncol..

[26] Christophi C., Nikfarjam M., Malcontenti-Wilson C., Muralidharan V. (2004). Long-term Survival of Patients with Unresectable Colorectal Liver Metastases treated by Percutaneous Interstitial Laser Thermotherapy. World J. Surg..

[27] Windahl T., Skeppner E., Andersson S.-O., Fugl-Meyer K.S. (2004). Sexual function and satisfaction in men after laser treatment for penile carcinoma. J. Urol..

[28] Lont A.P., Gallee M.P.W., Meinhardt W., van Tinteren H., Horenblas S. (2006). Penis Conserving Treatment for T1 and T2 Penile Carcinoma: Clinical Implications of a Local Recurrence. J. Urol..

[29] Meijer R.P., Boon T.A., van Venrooij G.E., Wijburg C.J. (2007). Long-Term Follow-up After Laser Therapy for Penile Carcinoma. Urology.

[30] Schlenker B., Tilki D., Seitz M., Bader M.J., Reich O., Schneede P., Hungerhuber E., Stief C.G., Gratzke C. (2010). Organ-preserving neodymium-yttrium-aluminium-garnet laser therapy for penile carcinoma: A long-term follow-up. BJU Int..

[31] Beer M., Jocham D., Beer A., Staehler G. (1989). Adjuvant Laser Treatment of Bladder Cancer: 8 Years’ Experience with the Nd-YAG Laser 1064 nm. BJU Int..

[32] Ruiz-Tovar J., González R., Conde S., Morales V., Martinez-Molina E. (2008). Jejunal and Bladder Perforation: Complication of Intravesical Nd-YAG Laser Irradiation of Bladder Tumour. Acta Chir. Belg..

[33] Beisland H.O., Sander S., Fossberg E. (1985). Neodymium:Yag laser irradiation of urinary bladder tumors Follow-up study of 100 consecutively treated patients. Urology.

[34] Kardos R., Magasi P., Karsza A. (1994). Nd-Yag laser treatment of bladder tumours. Int. Urol. Nephrol..

[35] Cavaliere S., Foccoli P., Toninelli C., Feijo S. (1994). Nd:YAG laser therapy in lung cancer: An 11-year experience with 2,253 applications in 1,585 patients. J. Bronchol. Int. Pulmonol..

[36] Schwarzmaier H.-J., Eickmeyer F., von Tempelhoff W., Fiedler V.U., Niehoff H., Ulrich S.D., Yang Q., Ulrich F. (2006). MR-guided laser-induced interstitial thermotherapy of recurrent glioblastoma multiforme: Preliminary results in 16 patients. Eur. J. Radiol..

[37] Streitparth F., Gebauer B., Melcher I., Schaser K., Philipp C., Rump J., Hamm B., Teichgräber U. (2009). MR-Guided Laser Ablation of Osteoid Osteoma in an Open High-Field System (1.0 T). Cardiovasc. Intervent. Radiol..

[38] Dick E.A., Joarder R., De Jode M.G., Wragg P., Vale J.A., Gedroyc W.M.W. (2002). Magnetic resonance imaging-guided laser thermal ablation of renal tumours. BJU Int..

[39] Di Matteo F., Picconi F., Martino M., Pandolfi M., Pacella C., Schena E., Costamagna G. (2014). Endoscopic ultrasound-guided Nd:YAG laser ablation of recurrent pancreatic neuroendocrine tumor: A promising revolution?. Endoscopy.

[40] Mauri G., Cova L., Ierace T., Baroli A., Di Mauro E., Pacella C.M., Goldberg S.N., Solbiati L. (2016). Treatment of Metastatic Lymph Nodes in the Neck from Papillary Thyroid Carcinoma with Percutaneous Laser Ablation. Cardiovasc. Intervent. Radiol..

[41] Vogl T.J., Straub R., Eichler K., Woitaschek D., Mack M.G. (2002). Malignant Liver Tumors Treated with MR Imaging–guided Laser-induced Thermotherapy: Experience with Complications in 899 Patients (2520 lesions). Radiology.

[42] Zukiwskyj M., Daly P., Chung E. (2013). Penile cancer and phallus preservation strategies: A review of current literature. BJU Int..

[43] Skeppner E., Windahl T., Andersson S.-O., Fugl-Meyer K.S. (2008). Treatment-Seeking, Aspects of Sexual Activity and Life Satisfaction in Men with Laser-Treated Penile Carcinoma. Eur. Urol..

[44] Hofstetter A.G. (1992). Application of lasers in bladder cancer. Semin. Surg. Oncol..

[45] Kramer M.W., Bach T., Wolters M., Imkamp F., Gross A.J., Kuczyk M.A., Merseburger A.S., Herrmann T.R.W. (2011). Current evidence for transurethral laser therapy of non-muscle invasive bladder cancer. World J. Urol..

[46] D’Hallewin M.A., Clays K., Persoons A., Baert L. (1989). Large-bowel perforation. A rare complication of intravesical Nd-YAG laser irradiation of bladder tumors. Urol. Int..

[47] Dohmoto M., Hünerbein M., Schlag P.M. (1996). Palliative endoscopie therapy of rectal carcinoma. Eur. J. Cancer.

[48] Sherwood L.A., Knowles G., Wilson R.G., Potter M.A. (2006). Retrospective review of laser therapy for palliation of colorectal tumours. Eur. J. Oncol. Nurs..

[49] Simoff M.J. (2011). Advances in Supportive and Palliative Care for Lung Cancer Patients.

[50] Schilling A., Böwering R., Keiditsch E. (1986). Use of the neodymium-YAG laser in the treatment of ureteral tumors and urethral condylomata acuminata. Clinical experience. Eur. Urol..

[51] Akimov A.B., Seregin V.E., Rusanov K.V., Tyurina E.G., Glushko T.A., Nevzorov V.P., Nevzorova O.F., Akimova E.V. (1998). Nd:YAG interstitial laser thermotherapy in the treatment of breast cancer. Lasers Surg. Med..

[52] Johnson D.E. (1994). Use of the holmium:YAG (Ho:YAG) laser for treatment of superficial bladder carcinoma. Lasers Surg. Med..

[53] Razvi H.A., Chun S.S., Denstedt J.D., Sales J.L. (1995). Soft-Tissue Applications of the Holmium:YAG Laser in Urology. J. Endourol..

[54] Kramer M.W., Wolters M., Cash H., Jutzi S., Imkamp F., Kuczyk M.A., Merseburger A.S., Herrmann T.R.W. (2015). Current evidence of transurethral Ho:YAG and Tm:YAG treatment of bladder cancer: Update. World J. Urol..

[55] Syed H.A., Biyani C.S., Bryan N., Brough S.J.S., Powell C.S. (2001). Holmium:YAG Laser Treatment of Recurrent Superficial Bladder Carcinoma: Initial Clinical Experience. J. Endourol..

[56] Das A., Gilling P., Fraundorfer M. (1998). Holmium laser resection of bladder tumors (HoLRBT). Tech. Urol..

[57] Jønler M., Lund L., Bisballe S. (2004). Holmium:YAG laser vaporization of recurrent papillary tumours of the bladder under local anaesthesia. BJU Int..

[58] Hossain M.Z., Khan S.A., Salam M.A., Hossain S., Islam R. (2005). Holmium YAG laser treatment of superficial bladder carcinoma. Mymensingh Med. J..

[59] Zhu Y., Jiang X., Zhang J., Chen W., Shi B., Xu Z. (2008). Safety and Efficacy of Holmium Laser Resection for Primary Nonmuscle-Invasive Bladder Cancer Versus Transurethral Electroresection: Single-Center Experience. Urology.

[60] Wong K.A., Zisengwe G., Athanasiou T., O’Brien T., Thomas K. (2013). Outpatient laser ablation of non-muscle-invasive bladder cancer: Is it safe, tolerable and cost-effective?. BJU Int..

[61] Xishuang S., Deyong Y., Xiangyu C., Tao J., Quanlin L., Hongwei G., Jibin Y., Dongjun W., Zhongzhou H., Jianbo W. (2010). Comparing the safety and efficiency of conventional monopolar, plasmakinetic, and holmium laser transurethral resection of primary non-muscle invasive bladder cancer. J. Endourol..

[62] Matsuoka K., lida S., Tomiyasu K., Inoue M., Noda S. (2003). Transurethral endoscopic treatment of upper urinary tract tumors using a holmium:YAG laser. Lasers Surg. Med..

[63] Lindner U., Weersink R.A., Haider M.A., Gertner M.R., Davidson S.R.H., Atri M., Wilson B.C., Fenster A., Trachtenberg J. (2009). Image Guided Photothermal Focal Therapy for Localized Prostate Cancer: Phase I Trial. J. Urol..

[64] Amin Z., Lees W.R., Bown S.G. (1993). Interstitial laser photocoagulation for the treatment of prostatic cancer. Br. J. Radiol..

[65] Atri M., Gertner M.R., Haider M.A., Weersink R.A., Trachtenberg J. (2009). Contrast-enhanced ultrasonography for real-time monitoring of interstitial laser thermal therapy in the focal treatment of prostate cancer. Can. Urol. Assoc. J..

[66] Gangi A., Alizadeh H., Wong L., Buy X., Dietemann J.-L., Roy C. (2007). Osteoid Osteoma: Percutaneous Laser Ablation and Follow-up in 114 Patients. Radiology.

[67] Carpentier A., McNichols R.J., Stafford R.J., Itzcovitz J., Guichard J.-P., Reizine D., Delaloge S., Vicaut E., Payen D., Gowda A. (2008). Real-time magnetic resonance-guided laser thermal therapy for focal metastatic brain tumors. Neurosurgery.

[68] Dowlatshahi K., Francescatti D.S., Bloom K.J. (2002). Laser therapy for small breast cancers. Am. J. Surg..

[69] Haraldsdóttir K.H., Ivarsson K., Götberg S., Ingvar C., Stenram U., Tranberg K.-G. (2008). Interstitial laser thermotherapy (ILT) of breast cancer. Eur. J. Surg. Oncol..

[70] Gillams A.R., Lees W.R. (2000). Survival after percutaneous, image-guided, thermal ablation of hepatic metastases from colorectal cancer. Dis. Colon Rectum.

[71] Paulides M.M., Stauffer P.R., Neufeld E., Maccarini P.F., Kyriakou A., Canters R.A.M., Diederich C.J., Bakker J.F., Van Rhoon G.C. (2013). Simulation techniques in hyperthermia treatment planning. Int. J. Hyperth..

[72] Bruggmoser G. (2012). Some aspects of quality management in deep regional hyperthermia. Int. J. Hyperth..

[73] Rijnen Z., Bakker J.F., Canters R.A.M., Togni P., Verduijn G.M., Levendag P.C., Van Rhoon G.C., Paulides M.M. (2013). Clinical integration of software tool VEDO for adaptive and quantitative application of phased array hyperthermia in the head and neck. Int. J. Hyperth..

[74] Kok H.P., Kotte A.N., Crezee J. (2017). Planning, optimisation and evaluation of hyperthermia treatments. Int. J. Hyperth..

[75] Gellermann J., Wust P., Stalling D., Seebass M., Nadobny J., Beck R., Hege H.-C., Deuflhard P., Felix R. (2000). Clinical evaluation and verification of the hyperthermia treatment planning system hyperplan. Int. J. Radiat. Oncol..

[76] Verhaart R.F., Verduijn G.M., Fortunati V., Rijnen Z., van Walsum T., Veenland J.F., Paulides M.M. (2015). Accurate 3D temperature dosimetry during hyperthermia therapy by combining invasive measurements and patient-specific simulations. Int. J. Hyperth..

[77] Sreenivasa G., Gellermann J., Rau B., Nadobny J., Schlag P., Deuflhard P., Felix R., Wust P. (2003). Clinical use of the hyperthermia treatment planning system HyperPlan to predict effectiveness and toxicity. Int. J. Radiat. Oncol..

[78] Datta N.R., Ordóñez S.G., Gaipl U.S., Paulides M.M., Crezee H., Gellermann J., Marder D., Puric E., Bodis S. (2015). Local hyperthermia combined with radiotherapy and-/or chemotherapy: Recent advances and promises for the future. Cancer Treat. Rev..

[79] Crezee H., van Leeuwen C.M., Oei A.L., Stalpers L.J.A., Bel A., Franken N.A., Kok H.P. (2016). Thermoradiotherapy planning: Integration in routine clinical practice. Int. J. Hyperth..

[80] Kok H., Wust P., Stauffer P., Bardati F., van Rhoon G., Crezee J. (2015). Current state of the art of regional hyperthermia treatment planning: A review. Radiat. Oncol..

[81] Dewhirst M.W., Viglianti B.L., Lora-Michiels M., Hanson M., Hoopes P.J. (2003). Basic principles of thermal dosimetry and thermal thresholds for tissue damage from hyperthermia. Int. J. Hyperth..

[82] Saccomandi P., Schena E., Silvestri S. (2013). Techniques for temperature monitoring during laser-induced thermotherapy: An overview. Int. J. Hyperth..

[83] Ukimura O., de Castro Abreu A.L., Hung A.J., Gill I.S. (2014). Cryosurgery for clinical T3 prostate cancer. BJU Int..

[84] Schena E., Majocchi L. (2014). Assessment of temperature measurement error and its correction during Nd:YAG laser ablation in porcine pancreas. Int. J. Hyperth..

[85] Mocan L., Tabaran F.A., Mocan T., Bele C., Orza A.I., Lucan C., Stiufiuc R., Manaila I., Iulia F., Dana I. (2011). Selective ex vivo photothermal ablation of human pancreatic cancer with albumin functionalized multiwalled carbon nanotubes. Int. J. Nanomedicine.

[86] Matsumoto R., Selig A.M., Colucci V.M., Jolesz F.A. (1992). Interstitial Nd:YAG laser ablation in normal rabbit liver: Trial to maximize the size of laser-induced lesions. Lasers Surg. Med..

[87] Haynes M., Stang J., Moghaddam M. (2014). Real-time Microwave Imaging of Differential Temperature for Thermal Therapy Monitoring. IEEE Trans. Biomed. Eng..

[88] Marien A., Gill I., Ukimura O., Nacim B., Villers A. (2014). Target ablation—Image-guided therapy in prostate cancer. Urol. Oncol. Semin. Orig. Investig..

[89] Manns F., Milne P.J., Gonzalez-Cirre X., Denham D.B., Parel J.M., Robinson D.S. (1998). In situ temperature measurements with thermocouple probes during laser interstitial thermotherapy (LITT): Quantification and correction of a measurement artifact. Lasers Surg. Med..

[90] Van Nimwegen S.A., L’Eplattenier H.F., Rem A.I., van der Lugt J.J., Kirpensteijn J. (2009). Nd:YAG surgical laser effects in canine prostate tissue: Temperature and damage distribution. Phys. Med. Biol..

[91] Reid A.D., Gertner M.R., Sherar M.D. (2001). Temperature measurement artefacts of thermocouples and fluoroptic probes during laser irradiation at 810 nm. Phys. Med. Biol..

[92] Di Matteo F., Martino M., Rea R., Pandolfi M., Panzera F., Stigliano E., Schena E., Saccomandi P., Silvestri S., Pacella C.M. (2013). US-guided application of Nd:YAG laser in porcine pancreatic tissue: An ex vivo study and numerical simulation. Gastrointest. Endosc..

[93] Schena E., Tosi D., Saccomandi P., Lewis E., Kim T. (2016). Fiber Optic Sensors for Temperature Monitoring during Thermal Treatments: An Overview. Sensors.

[94] Taffoni F., Formica D., Saccomandi P., Di Pino G., Schena E. (2013). Optical fiber-based MR-compatible sensors for medical applications: An overview. Sensors.

[95] Cavaiola C., Saccomandi P., Massaroni C., Tosi D., Giurazza F., Frauenfelder G., Beomonte Zobel B., Di Matteo F.M., Caponero M.A., Polimadei A. (2016). Error of a Temperature Probe for Cancer Ablation Monitoring Caused by Respiratory Movements: Ex vivo and in vivo analysis. IEEE Sens. J..

[96] Polito D., Caponero M.A., Polimadei A., Saccomandi P., Massaroni C., Silvestri S., Schena E. (2015). A needle-like probe for temperature monitoring during laser ablation based on FBG: Manufacturing and characterization. J. Med. Device.

[97] Cappelli S., Saccomandi P., Massaroni C., Polimadei A., Silvestri S., Caponero M.A., Frauenfelder G., Schena E. Magnetic Resonance-compatible needle-like probe based on Bragg grating technology for measuring temperature during Laser Ablation. Proceedings of the 2015 37th IEEE Annual International Conference of the IEEE Engineering in Medicine and Biology Society (EMBC).

[98] Rieke V., Pauly K.B. (2008). MR thermometry. J. Magn. Reson. Imaging.

[99] Allegretti G., Saccomandi P., Giurazza F., Caponero M.A., Frauenfelder G., Di Matteo F.M., Beomonte Zobel B., Silvestri S., Schena E. (2015). Magnetic resonance-based thermometry during laser ablation on ex vivo swine pancreas and liver. Med. Eng. Phys..

[100] Vogl T.J., Straub R., Zangos S., Mack M.G., Eichler K. (2004). MR-guided laser-induced thermotherapy (LITT) of liver tumours: Experimental and clinical data. Int. J. Hyperth..

[101] Vogl T.J., Müller P.K., Hammerstingl R., Weinhold N., Mack M.G., Philipp C., Deimling M., Beuthan J., Pegios W., Riess H. (1995). Malignant liver tumors treated with MR imaging-guided laser-induced thermotherapy: Technique and prospective results. Radiology.

[102] Natarajan S., Raman S., Priester A.M., Garritano J., Margolis D.J.A., Lieu P., Macairan M.L., Huang J., Grundfest W., Marks L.S. (2016). Focal Laser Ablation of Prostate Cancer: Phase I Clinical Trial. J. Urol..

[103] Todd N., Diakite M., Payne A., Parker D.L. (2014). In vivo evaluation of multi-echo hybrid PRF/T1 approach for temperature monitoring during breast MR-guided focused ultrasound surgery treatments. Magn. Reson. Med..

[104] Bydder G.M., Kreel L. (1979). The temperature dependence of computed tomography attenuation values. J. Comput. Assist. Tomogr..

[105] Schena E., Saccomandi P., Giurazza F., Del Vescovo R., Mortato L., Martino M., Panzera F., Di Matteo F.M., Zobel B.B., Silvestri S. Monitoring of temperature increase and tissue vaporization during laser interstitial thermotherapy of ex vivo swine liver by computed tomography. Proceedings of the IEEE 2013 35th Annual International Conference of the IEEE Engineering in Medicine and Biology Society (EMBC).

[106] Pandeya G.D., Klaessens J.H., Greuter M.J., Schmidt B., Flohr T., Van Hillegersberg R., Oudkerk M. (2011). Feasibility of computed tomography based thermometry during interstitial laser heating in bovine liver. Eur. Radiol..

[107] Schena E., Saccomandi P., Giurazza F., Caponero M.A., Mortato L., Di Matteo F.M., Panzera F., Del Vescovo R., Beomonte Zobel B., Silvestri S. (2013). Experimental assessment of CT-based thermometry during laser ablation of porcine pancreas. Phys. Med. Biol..

[108] Fani F., Schena E., Saccomandi P., Silvestri S. (2014). CT-based thermometry: An overview. Int. J. Hyperth..

[109] Liguori C., Frauenfelder G., Massaroni C., Saccomandi P., Giurazza F., Pitocco F., Marano R., Schena E. (2015). Emerging clinical applications of computed tomography. Med. Devices.

[110] Weiss N., Sosna J., Goldberg S.N., Azhari H. (2014). Non-invasive temperature monitoring and hyperthermic injury onset detection using X-ray CT during HIFU thermal treatment in ex vivo fatty tissue. Int. J. Hyperth..

[111] Huang X., El-Sayed I.H., Qian W., El-Sayed M.A. (2006). Cancer cell imaging and photothermal therapy in the near-infrared region by using gold nanorods. J. Am. Chem. Soc..

[112] Mooney R., Schena E., Saccomandi P., Zhumkhawala A., Aboody K., Berlin J.M. (2017). Gold nanorod-mediated near-infrared laser ablation: In vivo experiments on mice and theoretical analysis at different settings. Int. J. Hyperth..

[113] MacLaughlin C.M., Ding L., Jin C., Cao P., Siddiqui I., Hwang D.M., Chen J., Wilson B.C., Zheng G., Hedley D.W. (2016). Porphysome nanoparticles for enhanced photothermal therapy in a patient-derived orthotopic pancreas xenograft cancer model: A pilot study. J. Biomed. Opt..

[114] Lin T., Yuan A., Zhao X., Lian H., Zhuang J., Chen W., Zhang Q., Liu G., Zhang S., Chen W. (2017). Self-assembled tumor-targeting hyaluronic acid nanoparticles for photothermal ablation in orthotopic bladder cancer. Acta Biomater..

[115] Mooney R., Schena E., Zhumkhawala A., Aboody K.S., Berlin J.M. Internal temperature increase during photothermal tumour ablation in mice using gold nanorods. Proceedings of the 2015 37th Annual International Conference of the IEEE Engineering in Medicine and Biology Society (EMBC).

[116] El-Sayed I.H., Huang X., El-Sayed M.A. (2006). Selective laser photo-thermal therapy of epithelial carcinoma using anti-EGFR antibody conjugated gold nanoparticles. Cancer Lett..

[117] Hirsch L.R., Stafford R.J., Bankson J.A., Sershen S.R., Rivera B., Price R.E., Hazle J.D., Halas N.J., West J.L. (2003). Nanoshell-mediated near-infrared thermal therapy of tumors under magnetic resonance guidance. Proc. Natl. Acad. Sci. USA.

[118] Brown S.C., Palazuelos M., Sharma P., Powers K.W., Roberts S.M., Grobmyer S.R., Moudgil B.M. (2010). Nanoparticle Characterization for Cancer Nanotechnology and Other Biological Applications. Cancer Nanotechnology: Methods and Protocols.

[119] Jain S., Hirst D.G., O’Sullivan J.M. (2012). Gold nanoparticles as novel agents for cancer therapy. Br. J. Radiol..

